# Correction to “Little MAO: Isolation of an {Al_4_} Methylalumoxane Species from the Reaction of Trimethylaluminum with Water”

**DOI:** 10.1021/acs.organomet.5c00266

**Published:** 2025-07-30

**Authors:** Tassilo Berger, Markus Kramer, Cäcilia Maichle-Mössmer, Reiner Anwander

The literature survey undertaken for our paper was incomplete, missing out on a highly relevant recent study by Korona et al.^1^

The authors of this paper used the stabilizing effect of additional nitrogen donor molecules L (L = pyridine, *p*-dimethylaminopyridine) to perform low-temperature (−78 °C) hydrolyses of AlMe_3_. The dimeric tetramethylalumoxane adducts [AlMe_2_(L)­(μ_3_-O)­AlMe_2_]_2_ could be obtained in high yields and their solid-state structures were determined. Such *N*-donor adducts are isostructural to the tetrahydrofuran adduct **1** reported in our paper.

We apologize for this oversight.
